# Reputation, How Obtained

**Published:** 1877-10

**Authors:** 

**Affiliations:** Uptonville


### Correspondence.

UPTONVILLE, Sept. 4th, 1877.

*Editors Medical Journal:*

At the conclusion of my last letter I promised to give you some account of my experience in the care of the pauper population. I now beg to be excused until another time, prefering at this time to detail how some of the medical profession at Uptonville gain

Five months time must be regarded as much too short to determine results. We have known similar disease to remain stationary seven years after removal, yet return to prove fatal at end of eight years. (ED.)

what they call a reputation. And at this point, let me call your attention to the inclosed editorial from the New York *Times*, of of September 9th, which seems to give an exact idea of the situation.

The prevailing idea of reputation seems to be, to have one's name constantly before the public, and hence it is impossible to scan one of our several daily papers without learning that Dr. A, B, C or D has made some wonderful operation upon some one, who, somehow, was the victim of a slight accident, or that Mr. F is rapidly convalescing from his recent severe illness, and thanks to the skill of Dr. K, will soon be about.

I spoke of the medical societies in this place. They too are made instrumental in gaining reputation. A report of a case of whooping-cough is presented by some member, and doubtless, much to his surprise (?) the morning papers announce that Dr.—— "read at the meeting of the Uptonville Pathological Society, last evening, a valuable paper on Pertussis," and the public are accordingly amazed at the exceeding wisdom of the members of said society, and of Dr.—— in particular.

Recently, a case of this mild milk and water method of dodging the code came under my observation. One of our really excellent societies was regaled with a long and verbose article, by Dr. Z, detailing his experience in the use of moon-shine in the treatment of various diseases. Dr. Z's article failed to create any excitement in the society, nor did any of the members think it necessary to point out any of the reader's many erroneous conclusions, all conceded to him the entire right and privilege of employing moonshine on all occasions, if he could gain any benefit to his patients (or patients to himself ) thereby.

You can judge of my surprise, however, when one of my patients asked me in a day or two, if I heard Dr. Z's great lecture before the Medical society the other evening. But I soon learned that Dr. Z's friends were reporting among the laity, the great success he was meeting with in the use of moon-shine, and the wonderful sensation his paper had created among the physicians, who saw, however, but little in the paper, save an advertising dodge.

The younger members of the profession who see those who are older, and consequently, those to whom they look for example, so eagerly crowding and jostling each other in their haste to gain reputation (notoriety) are a little apt to become discouraged in their endeavor to conform to the exact teachings of the code, and the great wonder is in view of the flaunting success of quackery, that more of the struggling generation of "young doctors" do not fall into the ways of charlatanism.

I beg leave to affix here the article from the *Times*, already alluded to, trusting that some of my medical brethren will be induced to see how out-siders look upon their petty schemes to out-run time, and gain a reputation in advance of experience, and upon insecure foundation of popular notoriety. The editorial is entitled "Dry-Nursing Reputation."

"Reputation, even of a moderate sort, is so desirable to have that it is entirely natural for men to make great and continuous efforts to secure it. It is their misfortune often that they confound reputation and notoriety, and labor for what they conceive to be fame by unbecoming and unremunerative means. In a democracy like ours honors are so much easier and plentier than in an aristocracy that there is always a struggle for them by persons unfit to wear them. A republic, whatever its excellencies, necessarily gives opportunities and encouragement to demagogues and pretenders, which they would not and could not have in a monarchy. Having accomplished a vast deal in a short time, we are prone to be over-eager for results. Many of us are so unhealthfully anxious to separate ourselves from the mass that we do not stop to reflect if the sort of separation we are pretty certain to get is worth the trouble and trick of getting it.

"Notoriety, however cheap, passes with the multitude for reputation, and the man who has gained it calls it by the better name. What he has contrived to get is reputation pure and simple; what many of his fellows have is mere notoriety. This is the true difference between the terms. Whether it be reputation or notoriety, few persons with an itching for it are disposed to let it take its own course. Afraid that it won't grow fast enough, they are ever ready, with all kinds of fertilizers and personal attention, to bring it forward. They force it in every way, and by their ceaseless forcing do it more harm than good, not unfrequently destroying it altogether. The number of men who act as dry-nurses to their own reputation in politics, literature, law, medicine, theology, and even in society, is much greater than the uninitiated suspect. Dry-nursing of this eccentric variety is so widely, diligently, regularly, and skillfully practiced that it well-nigh deserves to be ranked as a profession. The profession is not avowed, however; it could not be without interfering with its own success.

"The popular notion is that any sort of reputation is got without the least agency or co-operation of the getter. This man or that woman does something, and the doing is so remarkable and weighty that it attracts public attention, and is duly chronicled by the press. Very often, generally perhaps, the notion is correct; but there are many cases in which it is wholly false. Members of the dry-nurse class have not sufficient faith in public or even in private appreciation. Even before they have delivered themselves of some mighty effort they are industrious in telling the proper persons how mighty the effort is to be, and of discovering the most efficient means of self-advertising. After they have delivered themselves they move heaven and earth to insure ample notice of their performance; and though they may fail partially they seldom fail entirely. Exertion, whether in a noble or ignoble cause, is always fruitful.

"Very ordinary arguments of many a lawyer have been put forward as matchless forensic efforts through persistent manipulation of his own. Judges' decisions that were weak, partial, and unsustained by precedent have been lauded and their lofty integrity commended, because they had been permitted to judge the Judge. Easy operations or accidental recoveries are made to appear as extraordinary evidences of surgical or medical skill, for no other reason than that the physician has a chance to tell his own story, and tells it so much more skillfully than he makes his diagnosis. He proves himself not only a learned gentleman, who amuses the patient while Nature performs the cure; he demonstrates his talent at hiding facts and presenting fiction in their light. Clergymen have been known to disseminate self-complimentary paragraphs of their own composition among reporters, and yet entertain and reiterate the belief that genuine ability must make its way, independent of encouragement or appreciation. There are dry-nurses to reputation in all callings; but the reputations which need dry-nurses never last. A reputation that is worth anything, that has any sound foundation, must be nourished at the fount of Nature."

I wish every quack, either public or secret, could read and digest those lines.

I have already exceeded my limits, and hence draw to a close.

Yours, NIL DESPERANDUM.

